# Proteomic analysis response of rice (*Oryza sativa*) leaves to ultraviolet-B radiation stress

**DOI:** 10.3389/fpls.2022.871331

**Published:** 2022-09-15

**Authors:** Saroj Kumar Sah, Salah Jumaa, Jiaxu Li, K. Raja Reddy

**Affiliations:** ^1^Department of Biochemistry, Molecular Biology, Entomology, and Plant Pathology, Mississippi State University, Mississippi State, MS, United States; ^2^Department of Plant and Soil Sciences, Mississippi State University, Mississippi State, MS, United States

**Keywords:** *Oryza sativa*, ultraviolet-B stress, leaf-proteome, two-dimensional gel electrophoresis, mass spectrometry

## Abstract

Rice (*Oryza sativa*) is a human staple food and serves as a model organism for genetic and molecular studies. Few studies have been conducted to determine the effects of ultraviolet-B (UV-B) stress on rice. UV-B stress triggers morphological and physiological changes in plants. However, the underlying mechanisms governing these integrated responses are unknown. In this study, we conducted a proteomic response of rice leaves to UV-B stress using two-dimensional gel electrophoresis and identified the selected proteins by mass spectrometry analysis. Four levels of daily biologically effective UV-B radiation intensities were imposed to determine changes in protein accumulation in response to UV-B stress: 0 (control), 5, 10, and 15 kJ m^−2^ d^−1^in two cultivars, i.e., IR6 and REX. To mimic the natural environment, we conducted this experiment in Sunlit Soil-Plant-Atmosphere-Research (SPAR) chambers. Among the identified proteins, 11% of differentially expressed proteins were found in both cultivars. In the Rex cultivar, only 45% of proteins are differentially expressed, while only 27.5% were expressed in IR6. The results indicate that REX is more affected by UV-B stress than IR6 cultivars. The identified protein TSJT1 (spot 16) in both cultivars plays a crucial role in plant growth and development during stress treatment. Additionally, we found that UV-B stress altered many antioxidant enzymes associated with redox homeostasis and cell defense response. Another enzyme, the glyceraldehyde-3-phosphate dehydrogenase (GAPDH), has been identified as spot 15, which plays an essential role in glycolysis and cellular energy production. Another vital protein identified is glycosyl hydrolase (GH) as spot 9, which catalyzes the hydrolysis of glycosidic bonds in cell wall polymers and significantly affects cell wall architecture. Some identified proteins are related to photosynthesis, protein biosynthesis, signal transduction, and stress response. The findings of our study provide new insights into understanding how rice plants are tailored to UV-B stress *via* modulating the expression of UV-B responsive proteins, which will help develop superior rice breeds in the future to combat UV-B stress. Data are available *via* ProteomeXchange with identifier PXD032163.

## Introduction

Sunlight emits a broad spectrum of radiation: visible light, ultraviolet (UV), and infrared radiation, which reaches the Earth's surface (Kumar et al., 2016). Among these radiations, UV rays have direct and indirect effects at every level of the biological organization of plant molecules, affecting all ecosystems. The UV is divided into three different forms based on their wavelengths such as UV-A (320–400 nm), UV-B (280–320 nm), and UV-C (100–280 nm). Among them, UV-C is the most dangerous radiation, entirely absorbed by the ozone layer in the atmosphere. In contrast, UV-A radiation is unaffected, though such radiation does not harm plants. However, the UV-B radiation intensity is affected mainly by the thickness of the stratospheric ozone layer and is most harmful to plants (Sztatelman et al., 2015). The amount of UV-B radiation has increased due to stratospheric ozone (O3) depletion caused by anthropogenic chlorofluorocarbons. The increased doses of UV-B radiation cause severe biological effects on plants and other organisms. UV-B damages the cells at many levels, including altering DNA, proteins, and lipids (Brown and Jenkins, 2008). The UV-B also damages the photosynthetic pigments in plants because DNA and proteins can absorb the UV spectrum, declining the plants' photosynthetic efficiency (Wu et al., 2011). Besides these, a high level of UV-B enhances the production of reactive oxygen species (ROS), which activates stress signaling pathways (Brown and Jenkins, 2008). Mainly, UV-B radiation directly damages the DNA by forming dimers between adjacent pyrimidines in DNA strands, blocking DNA polymerase's progress and leading to cell death (Rastogi et al., 2010; Santos et al., 2012). Some genes are identified as UV-B dependent genes and are responsible for synthesizing UV screening compounds, DNA repairs, and activation of oxidative enzymes (Brosche and Strid, 2003). In addition, UV radiation causes downregulation of photosynthesis-related proteins, such as light-harvesting Chl a/b binding protein, whereas it upregulates protective proteins such as pathogen-related protein-1 (PR-1) at the mRNA level (Surplus et al., 1998). UV-B radiation also negatively affects leaf, hypocotyl, and seedling growth (Robson and Aphalo, 2012; Biever et al., 2014; Fina et al., 2017). The effects of UV-B have been studied in different crops like *Glycine max* (Liang et al., 2006; Peng and Zhou, 2009; Iii et al., 2013), *Triticum aestivum* (Agrawal and Rathore, 2007), *Hordeum vulgare* (Fedina et al., 2003), *Pisum sativum* (Qu et al., 2006), *Zea mays* (Casati et al., 2005; Reddy et al., 2013), and *Arabidopsis thaliana* (Sztatelman et al., 2015). Rice (*Oryza sativa* L.) is one of the most important staple food crops for human consumption and a model plant for genetic and molecular study. Some studies have been conducted on the physiological response of UV-B stress on rice (He et al., 1993; Dai et al., 1994; Roy and Roy, 2013); however, only a few studies (Du et al., 2011; Wu et al., 2011) have been conducted on the UV-B stress on protein levels. Therefore, the UV-B-induced proteomic study on rice will provide new insight into understanding the mechanisms underlying injurious and protective responses of crops during UV-B stress.

Nowadays, a proteomic approach to revealing the mechanisms of plants under stress has become the method of choice. Mass spectrometry is an essential tool in proteomic research (Li and Assmann, 2000). Innovation in mass spectrometry has significantly improved the study of protein structure and function. Especially when the genome sequence is unavailable, amino acid sequencing is required for protein identification. Amino acid sequence information can be determined by mass spectrometry, which allows homology searching and cloning or database identification of the corresponding gene (Shevchenko et al., 1997). In the past, Li and Assmann showed that the de novo peptide sequencing of a low-abundance broad bean (*Vicia faba*) protein isolated from two-dimensional gels illustrates the power of mass spectrometry for protein identification (Li and Assmann, 2000). In this study, the proteome analysis of the rice leaves was determined for two different varieties (*Japonica* REX and *Indica* IR6) irradiated by artificial UV-B radiation. IR6 and REX are more popular than other *Indica* and *Japonica* rice cultivars and are cultivated widely, especially in the United States. Both were considered tolerant stress to temperature and drought. Previously, we studied the effect of day/night temperatures on rubisco activity and electron transport in these two varieties (Singh et al., 2019). Moreover, our previous studies showed that IR6 performed very well under low and high temperatures (Reddy et al., 2021). Thus, we want to see how both these varieties perform under UV-B stress. Both varieties showed significant changes in the number and expression of various proteins using mass spectrometry analysis. Specific novel UV-B proteins were identified, and their role in UV-B stress was implicated. The findings of this study may provide new insights into understanding how rice plants adapt to UV-B stress by modulating the expression of UV-B responsive proteins and the relevant genes, which may provide information for cultivating superior rice breeds in the future.

## Materials and methods

### Plant materials and experimental conditions

This experiment used two varieties of rice: *Japonica* Rex and *Indica* IR6. The rice varieties were obtained from Dr. Ed Rodona, Rice Breeder, Delta Research and Extension Center, Stoneville, MS, USA. This study was conducted in sunlit Soil-Plant-Atmosphere-Research (SPAR) chambers located at the Rodney Foil Plant Research Center, Mississippi State University, Mississippi State (33°28′ N, 88°47′ W), USA. All the SPAR chambers are made up of a steel soil bin (1-m deep by 2 m long by 0.5 m wide) to accommodate the root system and a Plexiglas chamber (2.5 m tall by 2.5 m long by 1.5 m wide) to accommodate aerial plant parts, a heating and cooling system, and an environmental monitoring and control system. The Plexiglas allows 97% of the visible solar radiation to pass without spectral variability in absorption and is opaque to solar UV-B radiation (280–320 nm) but transmits 12% of UV-A radiation (wavelength 320–400 nm). The lamps were wrapped with pre-solarized 0.07 mm cellulose diacetate film (JCS Industries Inc., La Mirada, CA) to avoid the germicidal effects of UV-C radiation. During these experiments, the measured minimum, maximum, and mean of daily solar radiation were 7.96, 31.22, and 22.34 ± 0.63 MJ m^−2^ d^−1^, respectively. Temperatures in all SPAR units were maintained at 29/21°C (day/night) and CO_2_ 410 μmol mol^−1^ during the experimental period. Air temperature in all the SPAR units was documented and adjusted every 10 seconds within set points of 30/22°C (day/night) ± 0.5°C. The daytime temperature was initiated at sunrise and returned to the nighttime temperature 1 h after sunset (Reddy et al., 2013).

### Growth conditions and treatments

#### Plant culture

Two varieties of rice seeds (Rex and IR6) were sown on May 10, 2017, in polyvinyl chloride pots (6” diameter by 12” high) filled with sand with a layer of gravel at the bottom. Each pot had a small hole at the bottom for excess water drainage. A total of 48 pots were used in the study. Six replicates per treatment for each variety were used. For each treatment, different SPAR chambers were used. Six pots per cultivar were arranged as a completely randomized design in six rows with two pots per row in each SPAR chamber (Supplementary Figure 1). Initially, four seeds were sown in each pot, and 11 days after emergence, the plants were thinned to one plant per pot. To ensure promising water and nutrients for growth, plants were irrigated three times a day with full-strength Hoagland's nutrient solution delivered at 08:00, 12:00, and 17:00 h through an automated and computer-controlled drip system.

### UV-B radiation treatments

In the beginning, all the chambers were maintained at a 0 kJ m^−2^ d^−1^ UV-B level. Fifteen days after planting (15DAP), four levels of daily biologically effective UV-B radiation intensities, 0 (control), 5, 10, and 15 kJ m^−2^ d^−1^ were imposed and continued until harvest. The expected UV-B radiation dosage was provided by the square-wave UV-B supplementation system under near-ambient photosynthetically active radiation. The UV-B radiation was delivered daily from 08:00 to 16:00 h by eight fluorescent UV-313 lamps (Q-Panel Company, Cleveland, OH, USA) attached horizontally on a metal frame inside each chamber, powered by 40 W dimmable ballasts. However, the same setup was used in the control unit to simulate equal shading; however, the lamps were kept unilluminated. To filter out UV-C (<280 nm), we wrapped individual lamps with solarized 0.07 mm cellulose diacetate films (JCS Industries Inc., La Mirada, CA, USA), and these were replaced as needed to maintain the particular UV-B radiation treatment. The distance from the lamps to the plant canopy was maintained at 0.5 m and adjusted to match the plant height throughout the experiment. The anticipated UV-B level was supplied to the top of the plant canopy and was checked daily at 09:00 h with a UVX digital radiometer (UVP, Inc., San Gabriel, CA, USA) and standardized against an Optronic Laboratory Model 754 Spectroradiometer (Orlando, FL, USA) that was used initially to quantify lamp output. To ensure the plants received the exact UV-B dosage, we took the respective UV-B measurements at three locations, 50 cm apart, in each SPAR chamber. The measured average daily biologically effective UV-B radiation doses during the experimental period were 5.08 ± 0.13 for 5 KJ, 10.08 ± 0.14 for 10 kJ, and 15.9 ± 0.63 for 15 kJ of UV-B treatments.

#### Protein sample preparation

The second uppermost leaf of the treated rice plants was collected on the 20th day after UV-B exposure, control treatment, and 35 days after transplanting (35DAP). The samples were immediately frozen in liquid nitrogen and stored at −80°C for further use. Samples were ground to a fine powder in liquid nitrogen, and total proteins were extracted from rice leaves using the method of Wang et al. (2006), with slight modification. First, the 0.3–0.4 g fine powder was transferred into a 2 ml Eppendorf tube and filled with chilled 10% trichloroacetic acid (TCA)/acetone (Sigma-Aldrich, ≥ 99.0%). The mixture was vortexed and then centrifugated at 4°C for 3 mins at 16,000 x g, and the supernatant was discarded without touching the pellet with a glass pipette. The pellet was washed by adding 80% methanol plus 0.1 M ammonium acetate (Sigma, ~98%) and further washed with 80% acetone at 4°C for 3 mins at 16,000 × g to remove residual TCA and contaminants. The pellet was air-dried in the hood for at least 10 mins to remove residual acetone. The pellet was suspended in a 1:1 ratio of phenol (pH 8.0) and sodium dodecyl sulfate (SDS) buffer [30% sucrose, 2% SDS (Bio-Rad, 1610302), 0.1 M Tris-HCl] supplemented with 5% 2-mercaptoethanol (Thermo-Fisher) and vortex for 30 s and then incubated at room temperature for 5–10 mins. The mixture was centrifuged at 16,000 × g for 3 mins at room temperature. The upper phenol phase (about 300 μl) was transferred into a new 2-ml Eppendorf tube, filled with five volumes of cold methanol plus 0.1 M ammonium acetate, and incubated at −20°C for about 2 h. After incubation, the mixture was centrifuged at 16,000 × g for 5 mins at 4°C, and the supernatant was discarded without disturbing the pellet. The tube containing the pellet was filled with methanol plus 0.1 M ammonium acetate and centrifuged at 16,000 × g for 5 mins at 4°C. After centrifugation, the supernatant was discarded without disturbing the pellet. Finally, the pellet was washed with 80% acetone by centrifugation at 16,000 × g for 5 mins. The supernatant was discarded and pellet were air-dried to remove any remaining acetone, and the dried pellet (proteins) was stored in the tube at −80°C for further use.

### Protein solubilization and quantification

The protein pellet was resuspended in an appropriate volume (about 150 μl) of rehydration buffer [9% urea, 4% 3-[(3-cholamidopropyl)dimethylammonium]-1-propanesulfonate (CHAPS) (GoldBio, 75621033), 50 mM Dithiothreitol (DTT; GoldBio, 27565419) supplemented with BioLyte 3/10 Ampholyte (Bio-Rad, 1632094). After gentle mixing, the mixture was incubated for 1 h with agitation. The protein samples were centrifuged at 16,000 xg at room temperature for 3 mins. The protein content in the supernatant was quantified using the modified Bradford assay (Bradford, 1976).

### Two-dimensional gel (2-DE) electrophoresis

The 2-DE was performed using the precast immobilized pH gradient (IPG) strips (Bio-Rad Laboratories) in the first dimension by using an isoelectric focusing (IEF) system (PROTEAN IEF Cell, Bio-Rad Laboratories) according to the manufacturer's instructions. Rice leaf proteins (400 μg) in rehydration buffer (9 M urea, 4% CHAPS, 50 mM dithiothreitol, 0.1% Bio-Lyte Ampholyte) were added to individual channels of an IPG focusing tray (Bio-Rad Laboratories). The Bio-Rad IPG strips (pH 4–7, 11 cm) were then placed over the protein solutions in the channels of the IPG focusing tray. The protein samples were rehydrated in the IPG focusing tray at 50 Volt for 1 h. After rehydration, the samples were run at 250 Volt for 15 mins, then ramped to 8,000 Volt over 2.5 h, and kept at 8,000 Volt for 13 to 15 h. After isoelectric focusing, the IPG strips were equilibrated in equilibration buffer I (6 M urea, 0.375 M Tris-HCl, pH 8.8, 2% SDS, 20% glycerol, and 100 mM dithiothreitol) for 10 mins at room temperature and then placed in equilibration buffer II (100 mg Iodoacetamide, 6 M urea, 0.375 M Tris-HCl, pH 8.8, 2%SDS, 20% glycerol, and 0.01% bromophenol blue) for 10 mins. After incubation, the strips were directly placed on 12% polyacrylamide-SDS slab gels for second-dimension separation. Second-dimension gels were run at 8 mA per gel using a vertical gel electrophoresis system (R. Shadel Inc.). The same gel was stained with colloidal Coomassie blue or silver nitrate, and then the sensitivity in detecting different proteins between colloidal Coomassie staining and silver staining was compared. The results also showed that silver staining is more sensitive than colloidal Coomassie staining and provides better resolution of the spots (Supplementary Figure 2). After that, all the gels were then stained with silver staining, as described by Shevchenko et al. (1996), with minor modifications.

### Image analysis

After staining, the 2-DE images were obtained with a ChemiDoc Touch imaging system (Bio-Rad Laboratories), and protein spots were detected and analyzed with Image Lab 6.0 (Bio-Rad Laboratories). Silver-stained gels were stored in a 1% acetic acid solution at 4°C until further analysis.

### Protein digestion and mass spectrometry analysis

Protein spots on stained 2-DE gels were cut into ~1 mm^3^ pieces. Gel pieces were then subjected to a modified in-gel trypsin digestion procedure (Shevchenko et al., 1996). The samples were reduced by using DTT (1 mM concentration in 50 mM ammonium bicarbonate) for 30 mins at 60°C. The samples were then cooled to room temperature, and iodoacetamide (5 mM) was added for 15 mins in the dark at room temperature. DTT (5 mM) was then added to quench the reaction. Then sequence grade trypsin (Promega, Madison, WI) 5 ng/μl was added. After 45 mins, the excess trypsin solution was removed and replaced with a 50 mM ammonium bicarbonate solution to cover the gel pieces. The digestion was performed overnight at 37°C. Peptides were later extracted by removing the ammonium bicarbonate solution, followed by one wash with a solution containing 50% acetonitrile and 1% formic acid. The extracts were then dried in a speed-vac (~1 h). The samples were then stored at 4°C until analysis. On the analysis day, the samples were reconstituted in 5–10 μl of HPLC solvent A (2.5% acetonitrile, 0.1% formic acid). A nano-scale reverse-phase HPLC capillary column was created by packing 2.6 μm C18 spherical silica beads into a fused silica capillary (100 μm inner diameter × ~30 cm length) with a flame-drawn tip (Peng and Gygi, 2001). After equilibrating the column, each sample was loaded onto the column *via* a famous autosampler (LC Packings, San Francisco, CA). A gradient was formed, and peptides were eluted with increasing concentrations of solvent B (97.5% acetonitrile, 0.1% formic acid). As peptides were eluted, they were subjected to electrospray ionization and then entered into an LTQ Orbitrap Velos Pro ion-trap mass spectrometer (Thermo Fisher Scientific, Waltham, MA). Peptides were detected, isolated, and fragmented to produce a tandem mass spectrum of specific fragment ions for each peptide. Peptide sequences (and hence protein identity) were determined by matching protein databases (http://www.uniprot.org/proteomes/?query=taxonomy:39947) with the acquired fragmentation pattern by the software program, Sequest (Thermo Fisher Scientific, Waltham, MA) (Eng et al., 1994). All databases include a reversed version of all the sequences, and the data were filtered to between a one and two percent peptide false discovery rate.

### Functional analysis

Gene ontology(GO) annotation was performed *via* the Gene Ontology Database at (http://systemsbiology.cau.edu.cn/agriGOv2/) (Tian et al., 2017). The GO function of the identified proteins was classified into three categories, i.e., biological process, molecular process, and cellular component. The annotated GO term was plotted *via* a web tool, WEGO 2.0 (https://wego.genomics.cn/) (Ye et al., 2018). The pathway analysis was performed using the database RiceNetDB (http://bis.zju.edu.cn/ricenetdb/). The annotation was performed using the Kyoto Encyclopedia of Genes and Genomes (KEGG) (Liu et al., 2013). The gene functions were categorized based on databases, including the Rice Annotation Project Database (RAP-DB) build 5.0 (Sakai et al., 2013) by the International Rice Genome Sequencing Project (IRGSP), the MSU Rice Genome Annotation Database (Ouyang et al., 2007), and the Oryzabase Integrated Rice Science Database (https://shigen.nig.ac.jp/rice/oryzabase/).

## Results

### Morphological effects of UV-B on rice plants

**Figure 1:**
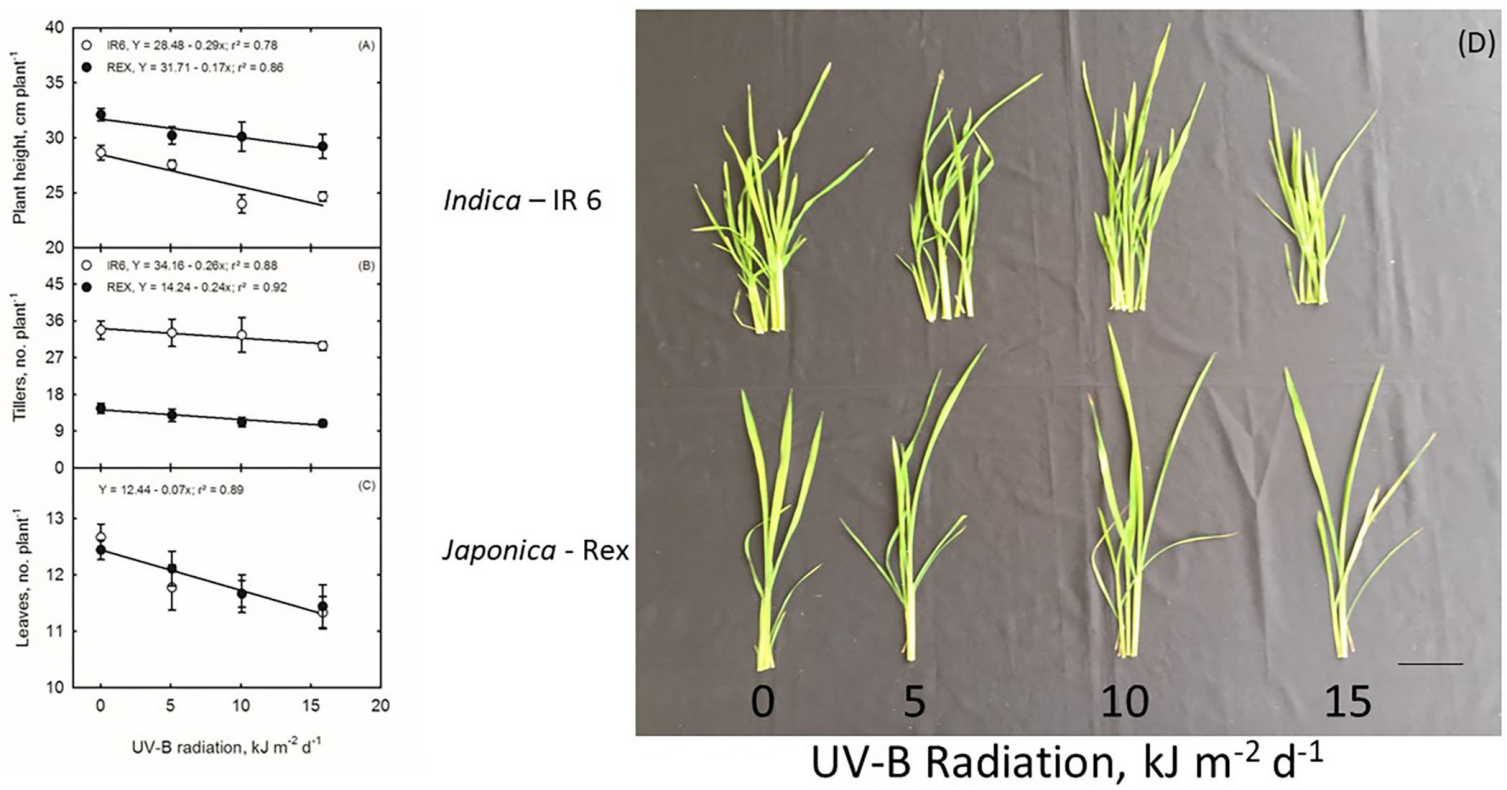
**(A–D)** Ultraviolet-B Effects on Rice Plants. Scale bar 2 cm.

In this study, the seedlings of two rice cultivars (REX, IR6) were treated with UV-B irradiation of 5, 10, and 15 kJ m^−2^ d^−1^ for 8 h daily. There is no morphological damage to plants, except for the fact that plants were shorter than the control (Figure 1). The plant height in both cultivars significantly differs between the treatments and cultivars. The UV-B effects on plant height in Rex cultivars are more severe than IR6 (Figure 1A). While increasing the UV-B dose, the plants become shorter in both varieties (Figure 1A). IR6 has more tillers than Rex, but there is no effect of UV-B stress on tiller number per plant in both cultivars compared to control (Figure 1B). The highly significant decreasing pattern in leaf numbers in both varieties is observed as UV-B stress dose increases compared to control (Figure 1C).

### Differentially expressed protein in rice cultivars under UV-B stress

**Figure 2:**
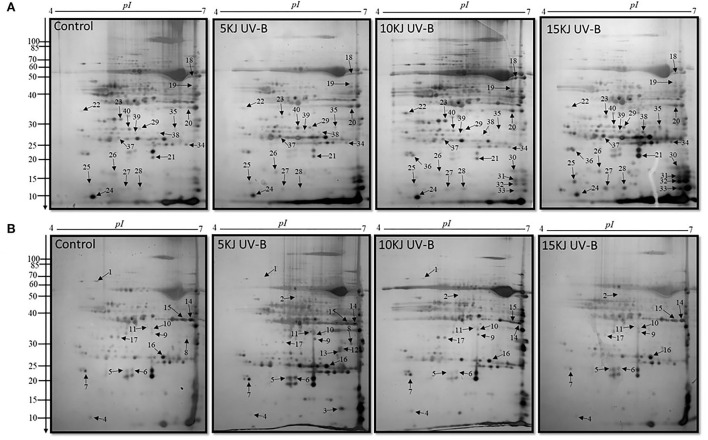
2D-GE maps of rice leaves proteins during 5, 10, and 15 kJ m^−2^ d^−1^ UV-B exposure along with control **(A)** cultivar REX **(B)** cultivar IR6. Quantitative image analysis revealed 40 differential spots were up and downregulated with silver staining. The black arrowheads showed the number of proteins identified. The numbers at the left of each panel are molecular masses in kilodaltons.

**Figure 3:**
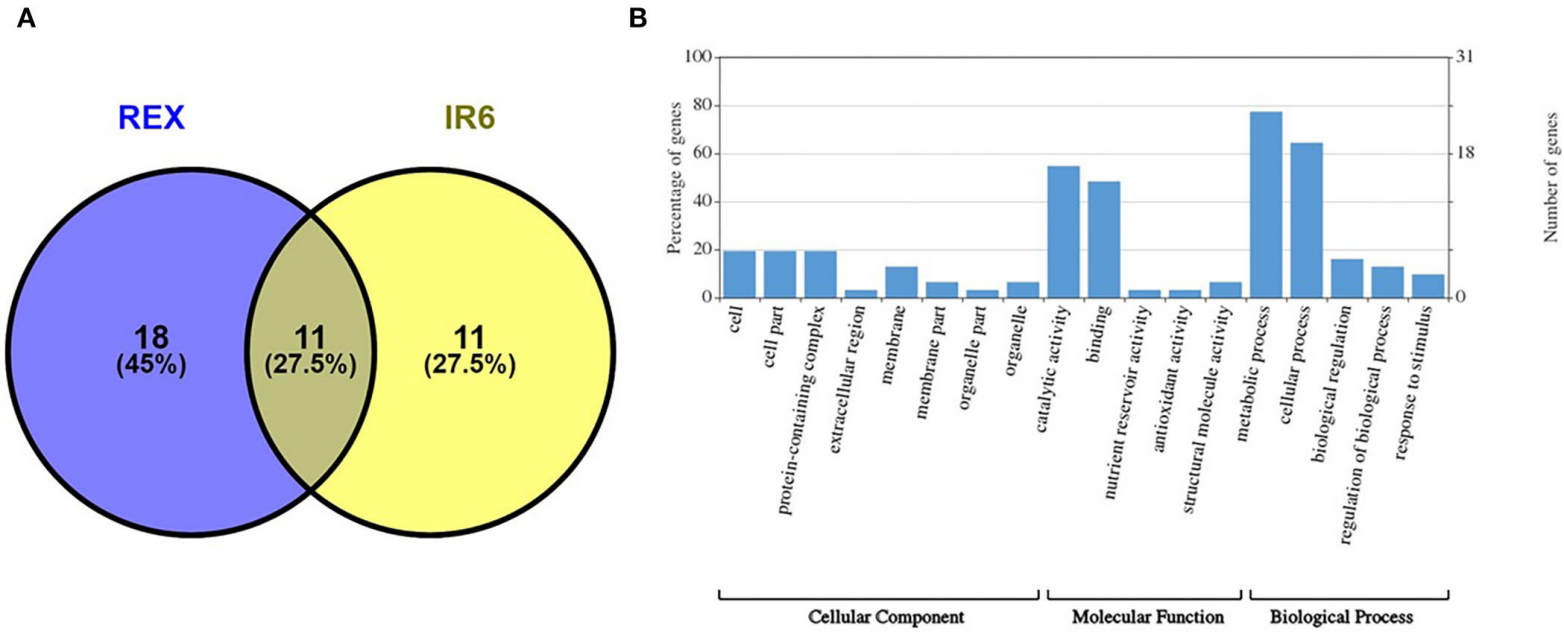
**(A)** Venn diagram showing the differentially expressed proteins in the cultivars REX and IR6 **(B)** GO enrichment analysis of identified proteins. The number of proteins enriched with each GO term in one of the three categories, including biological process, molecular function, and cellular component, is shown.

Proteomic analysis of rice leaves 2D-GE carried out the protein of two varieties modulated by UV-B stress. Each 2D-GE gel had ~1,000 protein spots covering a 4.0–7.0 pH range and a molecular weight of 4–120 KDa, based on their isoelectric points. Seventeen spots, labeled 1 to 17, showed significant changes in the IR6 varieties under three different levels of UV-B stress compared to their respective controls (Figure 2A). Similarly, 23 spots labeled 18 to 40 exhibited significant changes in the rice Rex varieties compared to control in UV-B stress (Figure 2B). Figure 2 shows the representative 2D-GE gel images of IR6 and Rex. By comparing the gels produced in the three different doses of UV-B treatment, the 18 spots in the IR6 variety displayed significant changes in expression. These protein spots exhibited altered expression profiles (1, 2, 4–11, 14–18, 21, 34, and 37; Figure 2) were successfully identified by mass spectrometry (Table 1). Similarly, there are 30 spots in Rex varieties in the different UV-B treatment, i.e., spot 1, 4–6, 14–16, 1–40 (Table 1). Among these spots, 11 spots were identified in both varieties. These spots were No. 1, 4–6, 14–16, 17, 21, 34, 37, and 40 (Table 1). Out of 11 spots, six spots (No. 4, 5, 14, 16, 21, and 37) showed a similar expression pattern in both the varieties in all the different levels of UV-B treatment (Table 2). Altogether, in both varieties, 40 spots were identified. The Venn diagram showed the differentially expressed proteins in both the cultivars (Figure 3A). A total of 45% of proteins were differentially expressed in the Rex cultivar, whereas only 27.5% were expressed in the IR6 cultivar. Moreover, 11% of differentially expressed proteins were common in both cultivars (Figure 3A). These results suggest that REX is more affected by UV-B stress than IR6 cultivars.

**Table 1:** List of proteins in rice leaves under UV-B stress identified by mass spectrometry.

**Spot no**.	**Protein description**	**Accession no**.	**pI**	**Mw**	**% Sequence coverage**	**FDR**
1	60 kDa chaperonin alpha subunit	LOC_Os12g17910.1	5.12	61,129.76	39.62	0.000
2	Histidinol dehydrogenase, chloroplast precursor	LOC_Os01g13190.1	6.03	50,566.08	9.51	0.000
3	Ubiquitin-conjugating enzyme, putative, expressed	LOC_Os12g41220.1	6.42	16,664.90	32.19	0.000
4	Glycine Cleavage system H proten, mitochondrial	LOC_Os10g37180.1	4.92	17,367.31	8.54	0.000
5	Germin-like protein 8-14/Cupin domain containing protein	LOC_Os08g35760.1	6.01	21,861.22	9.39	0.000
6	Glyoxalase family protein, expressed	LOC_Os05g22970.1	5.84	21,329.11	32.8	0.000
7	Peptidyl-prolyl cis-trans isomerase, putative expressed	LOC_Os02g02890.1	8.61	18,360.98	18.02	0.000
8	dehydrogenase, putative, expressed	LOC_Os01g53910.1	6.52	32,019.87	35.44	0.000
9	glycosyl hydrolase, putative, expressed	LOC_Os05g15770.1	6.08	32,548.96	13.13	0.000
10	26S proteasome non-ATPase regulatory subunit 14	LOC_Os01g16190.1	6.12	34,338.55	17.59	0.000
11	ubiquinone biosynthesis protein COQ9, mitochondrial precursor	LOC_Os03g29180.1	5.82	34,328.53	10.67	0.000
12	chlorophyll A-B binding protein, putative	LOC_Os01g64960.1	6.41	27,903.34	26.87	0.000
13	expressed protein	LOC_Os03g60740.1	6.34	27,910.55	31.13	0.000
14	Fructose-bisphosphate aldolase	LOC_Os08g02700.1	6.62	38,393.58	3.87	0.000
15	glyceraldehyde-3-phosphate dehydrogenase, putative, expressed	LOC_Os04g40950.1	6.34	36,772.98	6.53	0.000
16	Stem-specific protein TSJT1, putative, expressed	LOC_Os03g53270.1	6.05	26,432.92	25.9	0.000
17	oxygen-evolving enhancer protein 1, chloroplast precursor	LOC_Os01g31690.1	6.08	34,861.42	57.96	0.000
18	Photosystem II CP43 reaction center protein	LOC_Os04g16874.1	6.71	52,076.13	14.59	0.000
19	uncharacterized oxidoreductase, putative, expressed	LOC_Os10g26400.2	6.51	41,013.19	26.84	0.000
20	Peroxidase precursor, putative, expressed	LOC_Os03g22010.1	6.51	33,426.69	21.56	0.000
21	Ethylene-responsive protein-like	LOC_Os02g47840.1	5.96	19,874.78	19.21	0.000
22	gibberellin receptor, putative, expressed	LOC_Os09g28630.1	4.95	33,240.63	16.19	0.000
23	oxygen-evolving enhancer protein 1, chloroplast precursor	LOC_Os01g31690.1	6.08	34,861.42	63.36	0.000
24	glyoxalase family protein	LOC_Os02g17920.1	4.32	8,982.11	40.24	0.000
25	pathogenesis-related Bet v I family protein, putative, expressed	LOC_Os12g36880.1	4.88	16,687.92	13.92	0.000
26	Eukaryotic translation initiation factor 5A	LOC_Os07g02210.2	5.45	17,765.89	17.9	0.000
27	Glyoxalase family protein, expressed	LOC_Os03g45720.1	5.47	15,174.04	53.19	0.000
28	Superoxide dismutase [Cu-Zn], chloroplastic	LOC_Os08g44770.1	5.79	21,300.99	36.02	0.000
29	expressed protein	LOC_Os08g16570.1	5.89	31,852.54	42.52	0.000
30	osmotin, putative, expressed	LOC_Os12g38170.1	6.27	23,787.37	20.17	0.000
31	30S ribosomal protein S7, chloroplastic	LOC_Os10g21372.1	11.82	17,628.78	26.28	0.000
32	40S ribosomal protein S16	LOC_Os12g03090.1	10.58	16,818.90	27.52	0.000
33	40S ribosomal protein S19	LOC_Os03g31090.1	10.04	16,386.81	23.97	0.000
34	Cold shock domain protein 1	LOC_Os02g02870.1	6.64	22,723.36	12.03	0.000
35	chlorophyll A-B binding protein, putative, expressed	LOC_Os01g64960.1	6.41	27,903.34	11.19	0.000
36	ubiquitin-conjugating enzyme E2-22 kDa, putative, expressed	LOC_Os01g70140.1	4.95	21,323.19	26.15	0.000
37	Triosephosphate isomerase, cytosolic	LOC_Os01g05490.1	5.38	27,062.95	35.57	0.000
38	Proteasome subunit alpha type-3/peptidase, T1 family	LOC_Os01g59600.2	5.74	27,238.06	34.14	0.000
39	stem-specific protein TSJT1, putative, expressed	LOC_Os03g53270.1	6.05	26,432.92	20.72	0.000
40	chlorophyll A-B binding protein, putative, expressed	LOC_Os03g39610.1	5.62	28,495.40	15.97	0.000

Spot No. corresponds to the spot numbers on the gels in Figure 2. MW, Calculated molecular mass in kilodalton (kD) and pI, isoelectric point based on protein sequence annotated in the UniProt database.

**Table 2:** Functional classification and expression pattern of proteins in rice leaves under different levels of UV-B response.

**Functions**	**Spot no**.	**Protein spots description**	**Change pattern**				**Change pattern**			
			**in REX**				**in IR6**			
			**C**	**5**	**10**	**15**	**C**	**5**	**10**	**15**
Cell defense	11	Ubiquinone biosynthesis protein COQ9, mitochondrial precursor	ND	ND	ND	ND	+	↑	↑	↓
	20	Peroxidase precursor, putative, expressed	+	↑	↑	↑	ND	ND	ND	ND
Growth and development	16	Stem-specific protein TSJT1, putative, expressed	+	↑	↑	↑	+	↑	↑	↑
	39	Stem-specific protein TSJT1, putative, expressed	+	↑	↑	↑		ND	ND	ND
Metabolism	37	Triosephosphate isomerase, cytosolic	ND	↑	↑	↑	+	↑	↑	↑
	15	Glyceraldehyde-3-phosphate dehydrogenase, putative, expressed	+	↓	↓	↓	+	↓	↓	↑
	14	Fructose-bisphosphate aldolase	+	↓	↓	↓	+	↓	↓	↓
	8	Dehydrogenase, putative, expressed	ND	ND	ND	ND	+	↑	ND	ND
	13	NAD(P)-binding domain containing protein expressed protein	ND	ND	ND	ND	ND	+	ND	ND
	9	Clycosyl hydrolase, putative, expressed	ND	ND	ND	ND	+	↑	↑	↑
	19	Uncharacterized oxidoreductase, putative, expressed	ND	↑	↑	↓	ND	ND	ND	ND
Photosynthesis	35	Chlorophyll A-B binding protein, putative, expressed	+	↑	↓	↑	ND	ND	ND	ND
	12	Chlorophyll A-B binding protein, putative	ND	ND	ND	ND	ND	+	ND	ND
	40	Chlorophyll A-B binding protein, putative, expressed	+	↓	↓	↓	ND	ND	ND	ND
	18	Photosystem II CP43 reaction center protein	+	↑	↑	↓	+	↑	↑	↓
	17	Oxygen-evolving enhancer protein 1, chloroplast precursor	ND	ND	ND	ND	+	↓	↓	↓
	23	Oxygen-evolving enhancer protein 1, chloroplast precursor	ND	ND	ND	ND	+	↓	↓	↓
	1	60 kDa chaperonin alpha subunit	+	↑	↓	↓	+	↓	↓	ND
Protein biosynthesis	3	Ubiquitin-conjugating enzyme, putative, expressed	ND	ND	ND	ND		ND	ND	ND
	2	Histidinol dehydrogenase, chloroplast precursor	ND	ND	ND	ND	ND	↑	↓	↓
	4	Glycine Cleavage system H proten, mitochondrial	+	↓	↑	↓	+	↓	↑	↓
	31	30S ribosomal protein S7, chloroplastic	ND	ND	↑	↑	ND	ND	ND	ND
	32	40S ribosomal protein S16	ND	ND	↑	↑	ND	ND	ND	ND
	33	40S ribosomal protein S19	ND	ND	↑	↑	ND	ND	ND	ND
	36	Ubiquitin-conjugating enzyme E2-22 kDa, putative, expressed	ND	ND	↑	↑	ND	ND	ND	ND
Redox Homeostasis	28	Superoxide dismutase [Cu-Zn], chloroplastic	+	↑	↑	↑	ND	ND	ND	ND
	10	26S proteasome non-ATPase regulatory subunit 14	ND	ND	ND	ND	+	↑	↑	↑
	38	Proteasome subunit alpha type-3/peptidase, T1 family	+	↑	↓	↑		ND	ND	ND
	6	Glyoxalase family protein, expressed	+	↓	↓	↑	+	↑	↓	↑
	24	Glyoxalase family protein	+	↓	↑	↓	ND	ND	ND	ND
	25	Pathogenesis-related Bet v I family protein, putative, expressed	+	↑	↑	↑	ND	ND	ND	ND
	27	Glyoxalase family protein, expressed	+	↑	↓	↑	ND	ND	ND	ND
	30	Osmotin (pathogen-related protein 5), putative, expressed	ND	ND	↑	↑	ND	ND	ND	ND
Signal transduction	7	Peptidyl-prolyl cis-trans isomerase, putative expressed	ND	ND	ND	ND	+	↑	↑	↓
	26	Eukaryotic translation initiation factor 5A	+	↑	↓	↑	ND	ND	ND	ND
	34	Glycine-rich protein 2, putative expressed	+	↓	↓	↑	+	↑	↓	↓
Storage Protein	5	Germin-like protein 8-14/Cupin domain containing protein	+	↑	↓	↑	+	↑	↓	↑
Stress response	21	Universal stress protein domain containing protein	+	↑	↑	↑	+	↑	↑	↑
	22	Gibberellin receptor, putative, expressed	+	↑	↑	↑	ND	ND	ND	ND
	29	Expressed protein	+	↑	↑	↑	ND	ND	ND	ND

“ND” indicates not detected protein spots; “+” indicates “detected” protein spots in control; The up arrow “↑” indicates upregulated protein spots related to the control, and the down arrow “↓” indicates down-regulated protein spots related to the control.

### Functional classification of protein spots

Forty protein spots exhibited altered expression profiles in both rice varieties (Figure 2). Among them, spot 12, 35, 16, and 39 were the same protein (Table 1). We next conducted a functional analysis of all identified proteins by mass spectrometry. Go enrichment analysis showed that proteins are involved in the metabolic and cellular processes in the subcategory of biological processes (Figure 3B). In addition, most of the proteins are also associated with molecular functions such as catalytic activity and binding (Figure 3B).

We did a pathway analysis further to understand the potential roles and significance of identified proteins. The KEGG analysis showed that most of the proteins are involved in metabolic pathways (Supplementary Table 2), which suggests that UV-B stress has a potential role in metabolic pathway regulation. Moreover, the cellular component analysis showed that the identified proteins mainly resided in the cell, cell part, or as a part of protein-containing complexes (Figure 3B).

Most identified proteins are involved in metabolism, photosynthesis, protein biosynthesis, and redox homeostasis (Table 2). Few proteins—such as peptidyl-prolyl cis-trans isomerase, eukaryotic translation initiation factor 5A, glycin-rich protein two—were involved in signal transduction. Three proteins are detected, i.e., universal stress protein domain-containing protein, gibberellin receptor, and expressed protein (Table 2) implicated in stress response. There are 7 spots identified in metabolism (i.e., spots 37, 15, 14, 8,13, 9, and 19). Spots 37 is identified as a triosephosphate isomerase (TPI), a glycolytic enzyme that catalyzes the reversible interconversion of dihydroxyacetone phosphate to glyceraldehyde-3-phosphate. In both the cultivars, TPI is upregulated at 5, 10, and 15 kJ m^−2^ d^−1^ UV-B levels compared to control (Table 2). Another important protein is identified as spot 15, which is glyceraldehyde-3-phosphate dehydrogenase (GAPDH). GAPDH expression is decreased in both the cultivars under 5 and 10 kJ m^−2^ d^−1^ UV-B levels compared to control, whereas the expression is high in IR6 under 15 kJ m^−2^ d^−1^ UV-B levels compared to control. Similarly, fructose-bisphosphate aldolase, putative, is identified as spot 14, which is downregulated in both the cultivars under all the UV-B dose levels compared to control. Dehydrogenase and NAD(P) binding domain-containing protein were only detected in IR6 at 5 kJ m^−2^ d^−1^ UV-B levels compared to control. Similarly, glycosyl hydrolases (i.e., spot 9) are upregulated only in IR6 under all the UV-B dose levels compared to control. Likewise, spot 19 is identified as an uncharacterized oxidoreductase, upregulated in IR6 under all the UV-B dose levels compared to control (Table 2).

Like in metabolism, seven proteins have been identified in the photosynthesis process. In this functional category, the same protein identified in two spots (i.e., spots 12 and 35) belongs to the family of light-harvesting complex chlorophyll a/b-binding proteins. Spots 35 were only detected in REX, not in the IR6 cultivar. Under 5 and 15 kJ m^−2^ d^−1^ UV-B level, chlorophyll a/b-binding protein is upregulated in REX cultivars, whereas the expression is decreased under 10 kJ m^−2^ d^−1^ UV-B level. However, in IR6, this protein is detected at the 5 kJ m-2 d-1 UV-B level, whose expression is also upregulated like in another cultivar (Table 2). Similarly, another protein in the same family is identified as spot 40, only detected in the REX cultivar. Photosystem II, CP43 reaction center protein, is recognized as spot 18. It is upregulated in both the cultivars at 5 and 10 kJ m^−2^ d^−1^ UV-B levels as compared to the control. However, this protein is downregulated in both the cultivars at the 15 kJ m^−2^ d^−1^ UV-B level as compared to the control (Table 2). Oxygen-evolving enhancer protein 1 is identified as two spots, i.e., 17 and 23, and expression is decreased under all levels of UV-B dose as compared to the control and only detected in the REX cultivar (Table 2). The 60 kDa chaperonin alpha sub-unit is identified as spot 1 and is noticed in both cultivars. At the 5 kJ m^−2^ d^−1^ UV-B level, expression of chaperonin protein is high in Rex but decreased in IR6; however, at the 10 kJ m^−2^ d^−1^ UV-B level, expression of this protein was reduced in both the cultivars (Table 2). Three proteins have been identified in the stress response category, i.e., spots 21, 22, and 29, and the expression level is high under all levels of UV-B dose in the REX cultivar, whereas only spot 21 has been detected in the IR6 cultivar, whose expression is high at the 5 and 10 kJ m^−2^ d^−1^ UV-B level but low at the 15 kJ m^−2^ d^−1^ UV-B levels as compared to control. UV-B also played a significant role in plant growth and development. Two spots (i.e., spots 16 and 39) have been identified in this category as the same protein, which is stem-specific protein TSJT1. TSJT1 expression levels are increased in both cultivars, which may explain why the plants look shorter after the UV-B treatment. Likewise, in stress response, there are three proteins identified in the signal transduction category as spots 7, 26, and 34 (Table 2). Another category is redox homeostasis, in which there are eight proteins identified, i.e., spots 6, 10, 24, 25, 27, 28, 30, and 38. Only one protein identified in the storage protein category, spot 5, is recognized as a germin-like protein. In addition, seven proteins have been identified in the protein biosynthesis category. In these categories, ribosomal proteins are the most predominant proteins (Table 2).

## Discussion

Unlike studies conducted in greenhouses, this research is carried out in (SPAR) chambers, which are closer to the natural environment. In this study, two varieties were used to compare the three levels of UV-B stress (5, 10, and 15 kJ m^−2^ d^−1^) to control. In this work, we discovered 38 distinct proteins that were differentially expressed in both cultivars at different UV-B exposure levels compared to controls. We found 38 proteins, including previously known proteins. We also identified some novel proteins. According to identified proteins and their expression levels, the REX cultivar is more sensitive to UV-B damage than IR6 (Figures 2, 3A).

### Effect of UV-B on plant growth and development-related proteins

Our studies identified the TSJT1 protein as spot 16 in both cultivars. After applying the UV-B stress, the expression of TSJT1 increases in both the cultivars compared to the control. Only a few studies have been conducted on the TSJT1 protein. In plants, internode elongation is correlated with cell elongation and cell division. Hu and coworkers reported that TSJT1 might act as a negative regulator in castor internode development (Hu et al., 2016). Another study demonstrated that the abundance of TSJT1 was decreased in the dwarf variety Zhebi 100 compared to Zhebi 26. Zhebi 26 suggests that TSJT1 plays a role in node development. Additionally, the expression of the TSJT1 protein demonstrated higher drought stress in roots and leaves in two cassava cultivars—SC124 and Arg7—which were identified in proteomic analysis (Zhao et al., 2015). Our study also observed that the plant height decreases after applying UV-B stress, and TSJT1 expression increases as UV-B stress is imposed. These results imply that TSJT1 inhibits plant growth and development when exposed to stress.

### Effect of UV-B on cell defense and redox homeostasis-related proteins

Reactive oxygen species (ROS) play a dual role as critical regulators of growth, development, and defense pathways and toxic byproducts of aerobic metabolism (Mittler et al., 2004). The production of ROS is associated with the loosening of the plant cell wall during cell elongation. Rapid plant growth is characterized by an increase in antioxidation capacity since excessive ROS generation might damage cells (Cui et al., 2012; Hu et al., 2016). An alternative method, such as ROS scavenging by superoxide dismutase, can control ROS levels in plants (Goossens et al., 2003). In our study, UV-B stress caused changes in a number of antioxidation enzymes related to redox homeostasis and cell defense response proteins. In this study, UV-B stress treatment in cultivar REX increased the abundance of superoxide dismutase (spot 28), pathogenesis-related Bet V I family protein (spot 25), osmotin (pathogen-related protein 5) (spot 30), and peroxidase precursor (spot 20) as compared to control. Hu et al. (2016) also reported that—in the castor bin (high stalk variety Zhebi 100)—peroxidase precursor, and superoxide dismutase abundance were increased. The results presented above, including previously published data, suggest that the enhanced abundance of these proteins may also imply that the anti-oxidative defense system and resistance to UV-B stress are increased in cultivar REX as compared to control. These proteins were not detected in IR6.

### Effect of UV-B on metabolism-related proteins

Glyceraldehyde-3-phosphate dehydrogenase (GAPDH) is an important enzyme involved in glycolysis and cellular energy production associated with plant development (Giegé et al., 2003). The primary function of GAPDH is to catalyze the conversion of glyceraldehyde-3-phosphate to 1,3-bisphosphoglycerate within the glycolytic pathways; therefore, it plays a crucial role in energy metabolism (Voss et al., 2007). Voss et al. (2007) used GAPDH as a model protein to study oxidative damage caused by irradiation such as UV-A, UV-B, and infrared (IR). They showed that only UV-B irradiation was more aggressive and that UV-B acted on specific amino acids, such as arginine, proline, and tyrosine (Voss et al., 2007). Our study identified that GAPDH (spot 15) and fructose-bisphosphate aldolase (spot 14) were decreased under UV-B stress. This agrees with an earlier report on cyanobacteria under UV-B stress using two-dimensional gel electrophoresis and matrix-assisted laser desorption/ionization-time of flight mass spectrometry (MALDI-TOF MS) (Babele et al., 2015). The enzyme fructose-bisphosphate aldolase occupies a central position in glycolysis and gluconeogenesis (Ziveri et al., 2017). Fructose-1,6 bisphosphate aldolase is an essential enzyme that converts fructose-1, 6-bis phosphate to glyceraldehyde-3-phosphate and dihydroxyacetone 3-phosphate. Under the stress conditions, glyceraldehyde-3-phosphate and fructose-1, 6 bis phosphate may be converted to glucose-6-phosphate to carry out the pentose phosphate pathway for NADPH synthesis (Nakahara et al., 2003). This may explain why both enzymes were downregulated in our study during UV-B stress. Fructose-bisphosphate aldolase is essential for bacterial multiplication and participates in the control of host redox homeostasis and the inflammatory immune response (Ziveri et al., 2017). This study also identified triosephosphate isomerase (OsTPI1.1) as spot 37. Interestingly, we found that OsTPI1.1 is upregulated in both cultivars under UV-B stress. The triosephosphate isomerase enzyme catalyzes the interconversion of dihydroxyacetone phosphate (DHAP) and GAPDH during glycolysis and gluconeogenesis. OsTPI1.1 also modulates ROS production as a resistance mechanism against *Xanthomonas oryzae pv oryzae (Xoo)* (Liu et al., 2018). *Xoo* causes severe damage to rice production worldwide (Liu et al., 2018). The above results, including our finding, suggest that UV-B plays a role in metabolic pathways, cell defense, and transcriptional regulation.

### Effect of UV-B on cell wall architecture

Moreover, in this study, we identified glycosyl hydrolase (GH) as spot 9, whose expression has increased in IR6. However, GH was not detected in the REX cultivar. The primary function of GH is to catalyze the hydrolysis of glycosidic bonds in cell wall polymers and can have significant effects on cell wall architecture (Sharma et al., 2013). In rice, 437 GH genes are classified into 34 families. They identified 138 GH genes that are highly diverged between monocots and dicots, 57 of which have diverged further in rice as compared with four monocot genomes. They found two-thirds of GH genes in rice are upregulated in response to biotic and abiotic stress treatments (Sharma et al., 2013). In our study, GH is also upregulated, suggesting that UV-B stress treatment plays a role in abiotic and biotic stress adaptation and cell wall architecture. So far, GH has not been identified in the UV-B stress study. GH is the new protein we identified in UV-B stress treatment, suggesting that it may have a new function in cell wall architecture under UV-B stress.

### Effect of UV-B on photosynthesis-related proteins

Photosynthesis and protein biosynthesis have a significant function in plant growth and development. UV-B responses on plant species and even genotypes within species are different (Yuan et al., 2000). Under UV-B stress, *Oryza sativa* cultivar Lemont, produced more water-soluble proteins without any harmful effects on rice leaf photosynthetic properties, which suggests that it might be related to its UV-B tolerance. However, in the same dose, it caused damage to pea plants (He et al., 1993). The net photosynthesis rate increased under moderate UV-B irradiation in *Pseudotsuga menziesii* (Bassman et al., 2002). The photosynthesis rate of *Acorus calamus* L. (sweet flag) plants was increased under low UV-B treatment, which led to increased biomass (Kumari et al., 2009). Similarly, all proteins associated with photosynthetic processes—such as oxygen evolution complex protein 1, RuBisCO, and carbonic anhydrase—were altered in *Oryza sativa* (Wu et al., 2011). In our study also, photosynthesis-related proteins such as chlorophyll A-B binding protein (12,35,40), photosystem II CP43 reaction center protein (spot 18), and oxygen-evolving enhancer protein (spot 17 and 23) were altered. Chlorophyll A-B binding protein is only detected in the cultivar REX. Spot 35 increases under lower and high doses but decreases under moderate doses. However, spot 40 proteins decreased under all levels of UV-B stress. Some proteins are represented by more than one spot. These spots may represent different products of two very closely related genes or post-translational modifications of a single protein. We also find that the expression level is also different, suggesting that there are probably different isoforms regulated in opposite ways or that one isoform is post-translationally regulated (Casati et al., 2011). Chlorophyll A-B binding proteins are part of the light-harvesting complex (LHC). The LHC functions as a light receptor that captures and delivers excitation energy to photosystems I and II. Chlorophyll A-B binding proteins are the most abundant membrane proteins in nature (Xu et al., 2011). The overexpression of chlorophyll A-B binding proteins enhances the stomatal sensitivity to ABA, whereas downregulation of chlorophyll A-B reduces the responsiveness of stomatal movement to ABA, which results in a decrease in plant tolerance to drought stress in Arabidopsis (Xu et al., 2011).

Similarly, we also identified photosystem II C43 reaction center protein (spot 18). CP43 is one of the components of the core complex of photosystem II (PSII). PSII is a large pigment-protein complex embedded in the thylakoid membrane of all oxygenic photosynthetic organisms. This pigment-protein complex contains the PSII core antenna proteins CP47 and CP43. These two proteins are the most dominant and long-lived proteins compared to other core RC proteins—D1 and D2 (Weisz et al., 2019). CP43 is increased in low and middle doses of UV-B but decrease in high dose of UV-B in both cultivars. We also identified oxygen-evolving enhancer protein 1 (OEE1) (spots 17 and 23) in both cultivars. The OEE1 is decreased in the cultivar IR6 but not detected in the REX cultivar. This protein's primary function is to stabilize the manganese cluster, the primary water-splitting site. Under drought and heat stress, OEE1 interacts with a dnaK family protein (HSP70, 4332413) in maize (Hu et al., 2015). The expression of OEE1 is also altered upon cytokine treatment (Talla et al., 2016). Recently, Zhao et al. (2021) reported that photoinactivation of the OEE regulates the photosynthetic strategy of the seagrass *Zostera marina* (Zhao et al., 2021). These data showed that UV-B plays a role in photosynthesis and ABA signaling partly by modulating ROS homeostasis.

### Effect of UV-B on protein biosynthesis-related proteins

In protein biosynthesis, the ubiquitin-proteasome pathway is essential for regulating cell physiology and a cellular signaling system because it removes most abnormal and short-lived peptides and proteins. Ubiquitin-conjugating enzymes (E2) play a significant role in transporting ubiquitin from the ubiquitin-activating enzyme (E1) to the ubiquitin-ligase enzyme (E3) and the substrate (Liu et al., 2020). This study identified ubiquitin-conjugating enzymes (E2), which are upregulated under UV-B stress. However, ubiquitin-conjugating enzyme (UBC) gene family expression was reduced in rice (E et al., 2015). Jeon et al. (2012) isolated a novel E2 ubiquitin-conjugating enzyme from wild rice plants and found it highly expressed in leaves treated with salicylic acid and UV-B. And they also showed that UBC1 confers disease resistance and UV-B tolerance in transgenic Arabidopsis plants (Jeon et al., 2012). In our study, E2 enzymes were only identified in REX cultivars—not in IR6. The expression of E2 is higher than wild type under moderate and high doses of UV-B. These results suggest that the REX cultivar has more UV-B tolerance than IR6.

Similarly, we identified histidinol dehydrogenase (spot 2). Histidinol dehydrogenase is the first enzyme characterized by plants. Histidinol dehydrogenase enzyme catalyzes the NAD-linked four-electron dehydrogenase reaction leading from histidinol to His (Nagai and Scheidegger, 1991). Histidinol dehydrogenase was upregulated in transplastomic Arabidopsis lines, suggesting that histidine should be accumulated in the transplastomic lines (Stavridou et al., 2019). Glutamate can also be metabolized to produce histidine *via* reactions catalyzed by histidinol phosphate phosphatase and histidinol dehydrogenase (Stepansky and Leustek, 2006). These results suggest that histidinol dehydrogenase plays a role in protein biosynthesis, which is also affected by UV-B stress. In this study, we also identified 3 different ribosomal proteins. Ribosomal proteins are well-known for their role in mediating protein synthesis and maintaining the stability of the ribosomal complex. All three identified proteins, 30S ribosomal protein S7 (spot 31), 40S ribosomal protein S16 (spot 32), and S19 (spot 33), are upregulated under UV-B stress in cultivar REX. These three proteins are novel and have not been identified under UV-B stress. Some other ribosomal proteins in maize and Arabidopsis, such as RPL10A and RPL10C, were significantly upregulated under UV-B stress (Casati and Walbot, 2003; Ferreyra et al., 2010). These results suggest that UV-B plays a role in protein biosynthesis. Additionally, this study provides a resource for subsequent exploitation of RPL genes to improve abiotic and biotic stress conditions in rice and some other crops in the future.

### Effect of UV-B on signal transduction and stress response-related proteins

This study identified peptidyl-proyl cis-trans isomerase (PPIases) (spot 7). PPIases accelerate the folding of proteins. PPIases of rice OsIAA11 catalyzed by LATERAL ROOTLESS2 (LRT2), which is a cyclophilin-type peptidyl-prolyl cis/trans isomerase, directly regulates the stability of OsIAA11 (Jing et al., 2015). Knockdown of OsIAA11 expression partially rescues the LRT2 mutant phenotype in lateral root development. These results showed that cyclophilin-catalyzed peptidyl-prolyl isomerization promotes Aux/IAA degradation to regulate auxin signaling in rice (Jing et al., 2015; Acevedo et al., 2019). Plant cyclophilins have essential cellular functions in stress survival and the initiation of lateral root development (Kang et al., 2013). PPIase overexpressed under a low and moderate level of UV-B stress, whereas it was downregulated under a high level of UV-B doses in cultivar IR6 compared to control. So far, PPIase has not been identified in UV-B stress. Similarly, we also identified eukaryotic translation initiation factor 5A (eIF5A), which is upregulated in low and high doses of UV-B, whereas it was downregulated in the middle dose of UV-B. TaeIF5A1 from *Tamarix androssowii*, TaWRKY, and RAV protein share very similar expression patterns, and all stress-responsive genes are involved in the ABA signaling pathway (Wang et al., 2012). eIF5A-2 from Arabidopsis played a crucial role in plant growth and development by regulating cell division, cell growth, and cell death (Feng et al., 2007). This eIF5A has not been studied under UV-B stress. This study also identified glycine-rich protein 2, also called cold shock domain protein. In Arabidopsis, cold shock domain protein 2 negatively regulates seed germination by controlling ABA and GA levels (Sasaki et al., 2015). These results suggest that UV-B plays a role in signal transduction as well as the stress response.

**Figure 4:**
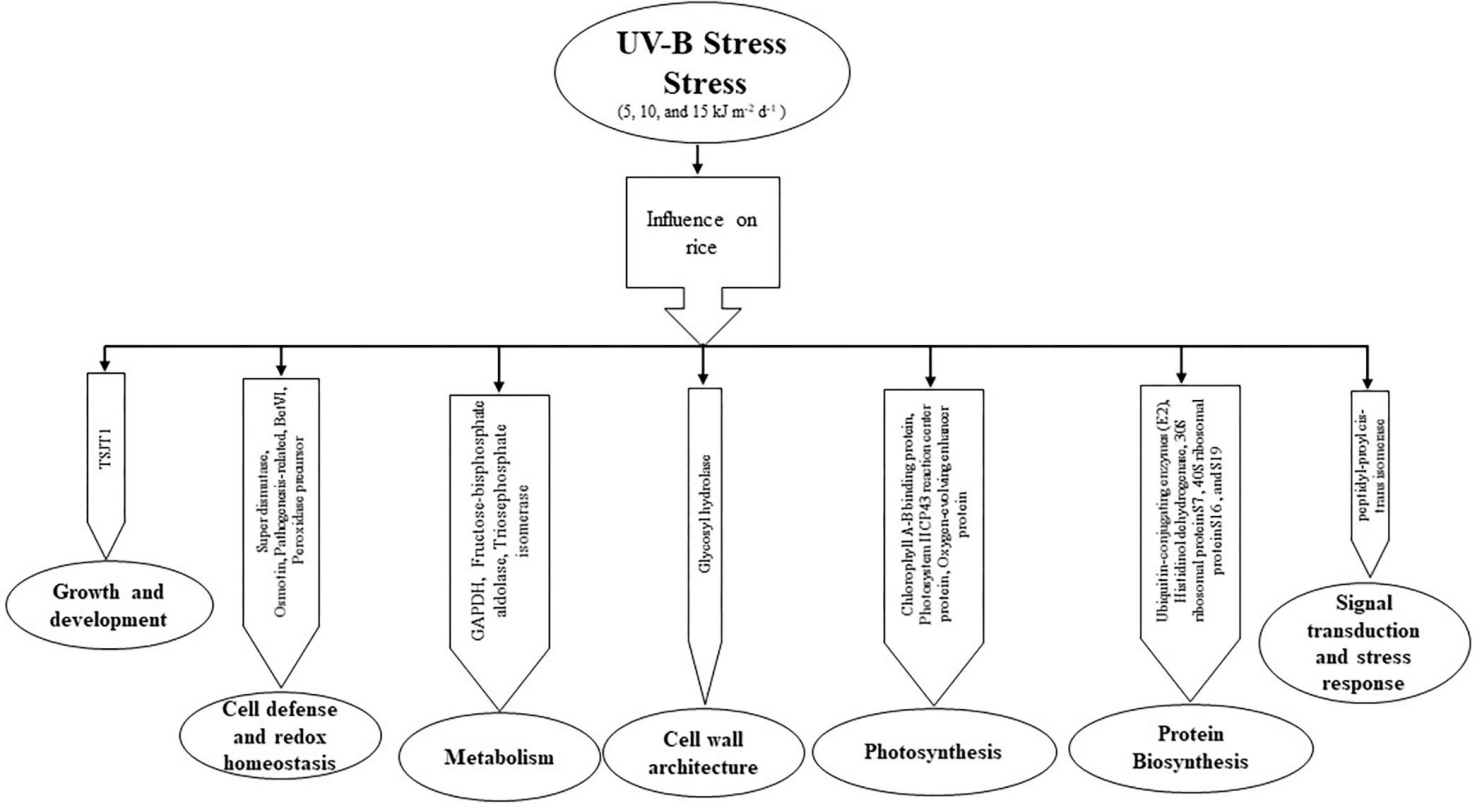
The pathway of UV-B induced protein expression in the proteomic response of rice leaves to cultivars IR6 and REX.

In conclusion, we found that protein expression under different levels of UV-B stress seems to differ in both cultivars. The REX cultivar is more affected by UV-B stress than the IR6 cultivar. The differentially expressed proteins are related to plant growth and development, cell defense and redox homeostasis, metabolism, cell wall architecture, photosynthesis, signal transduction, stress response, and ABA signaling (Figure 4). Morphological and physiological studies are further required to understand the function of this protein in detail. This study helped develop the molecular marker in rice's molecular breeding to enhance the production during excessive UV-B exposure.

## Data availability statement

The datasets presented in this study can be found in online repositories. The names of the repository/repositories and accession number(s) can be found below: The mass spectrometry proteomics data have been deposited to the ProteomeXchange Consortium *via* the PRIDE (Perez-Riverol et al., 2022) partner repository with the dataset identifier PXD032163.

## Author contributions

SS: conceptualization, data curation, formal analysis, methodology, visualization, and writing–original draft and editing. SJ: data collection and review. JL: supervision, funding, review, and editing. KR: conceptualization, methodology, supervision, funding, review, and editing. All authors have read and approved the manuscript.

## Funding

This research is partially funded by the National Institute of Food and Agriculture, NIFA 2019-34263-30552 and MIS 043050, and the MAFES SRI.

## Conflict of interest

The authors declare that the research was conducted in the absence of any commercial or financial relationships that could be construed as a potential conflict of interest.

## Publisher's note

All claims expressed in this article are solely those of the authors and do not necessarily represent those of their affiliated organizations, or those of the publisher, the editors and the reviewers. Any product that may be evaluated in this article, or claim that may be made by its manufacturer, is not guaranteed or endorsed by the publisher.

## Supplementary material

Supplementary Figure 1 Schematic illustration of the layout of experiments.

Supplementary Figure 2 Comparison of staining method in 2 D Gel analysis of control rice leaves samples **(A)** Colloidal coomassie staining and **(B)** Silver staining. The arrow indicates the protein spots detected only in silver staining, not coomassie staining.

Supplementary Table 1 GO enrichment analysis of identified proteins. The number of proteins enriched with each GO term in one of the three categories, including biological process, molecular function, and cellular component, is shown. The number in BG/RF refers to the annotated number in the background/reference. MSU7.0 gene ID (TIGR) was used as a reference.

Supplementary Table 2 Pathway analysis of identified proteins through mass spectrometry under different levels of UV-B stress.
