# Supplementary material for: Proteomic analysis response of rice (Oryza sativa) leaves to ultraviolet-B radiation stress
Source: Front Plant Sci. 2022 Sep 15;13:871331. doi: 10.3389/fpls.2022.871331 (PMC9536139; doi:10.3389/fpls.2022.871331)
Supplement: Supplementary Figure 1 — Schematic illustration of the layout of experiments. [file Image_1.pdf]

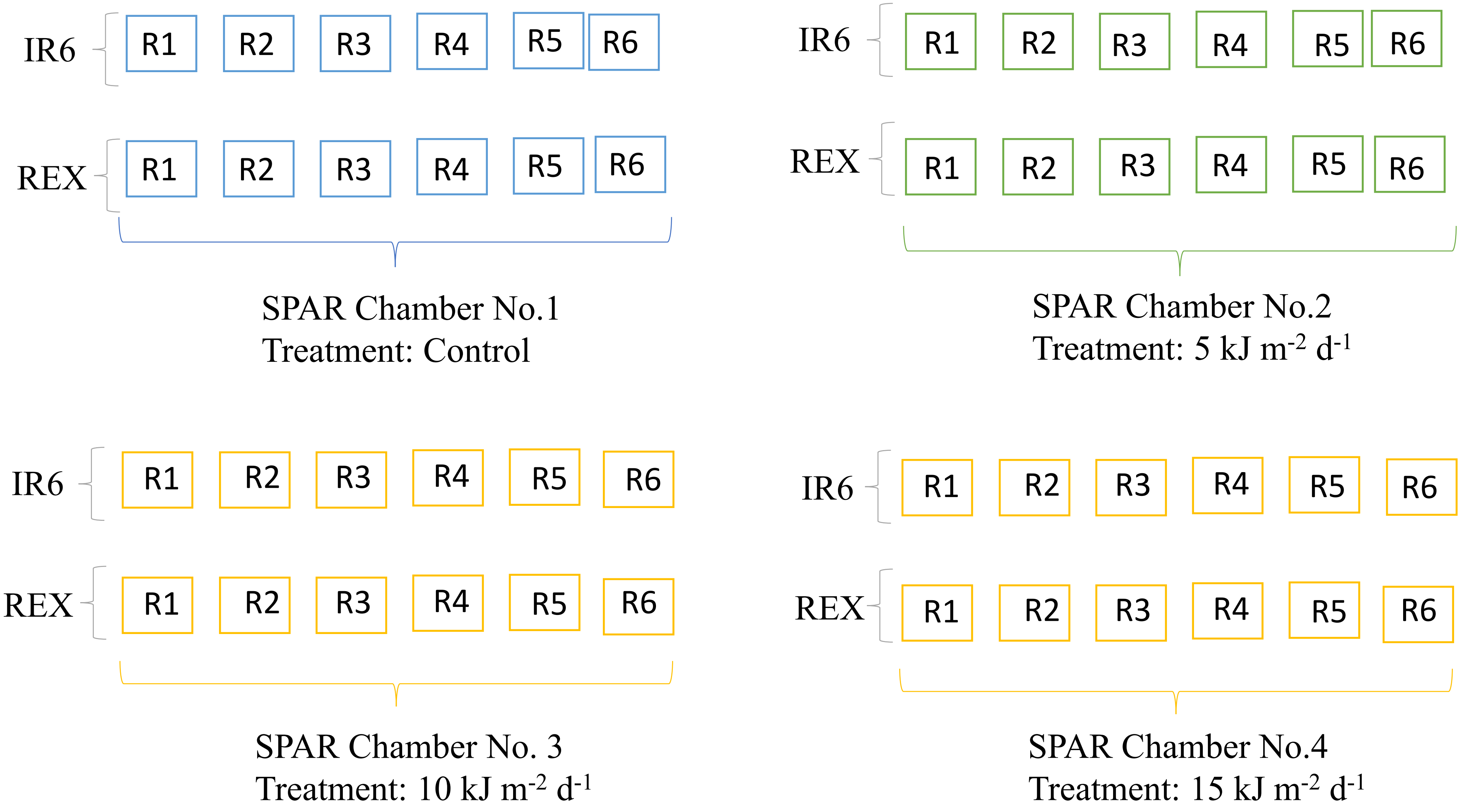
IR6
R1
R2
R3
R4
R5
R6
REX
R1
R2
R3
R4
R5
R6
SPAR Chamber No.1
Treatment: Control
IR6
R1
R2
R3
R4
R5
R6
REX
R1
R2
R3
R4
R5
R6
SPAR Chamber No.2
Treatment: 5 kJ m^{-2} d^{-1}
IR6
R1
R2
R3
R4
R5
R6
REX
R1
R2
R3
R4
R5
R6
SPAR Chamber No. 3
Treatment: 10 kJ m^{-2} d^{-1}
IR6
R1
R2
R3
R4
R5
R6
REX
R1
R2
R3
R4
R5
R6
SPAR Chamber No.4
Treatment: 15 kJ m^{-2} d^{-1}
