# Supplementary figures and images for: Proteomic analysis response of rice (Oryza sativa) leaves to ultraviolet-B radiation stress
Source: Front Plant Sci. 2022 Sep 15;13:871331. doi: 10.3389/fpls.2022.871331 (PMC9536139; doi:10.3389/fpls.2022.871331)

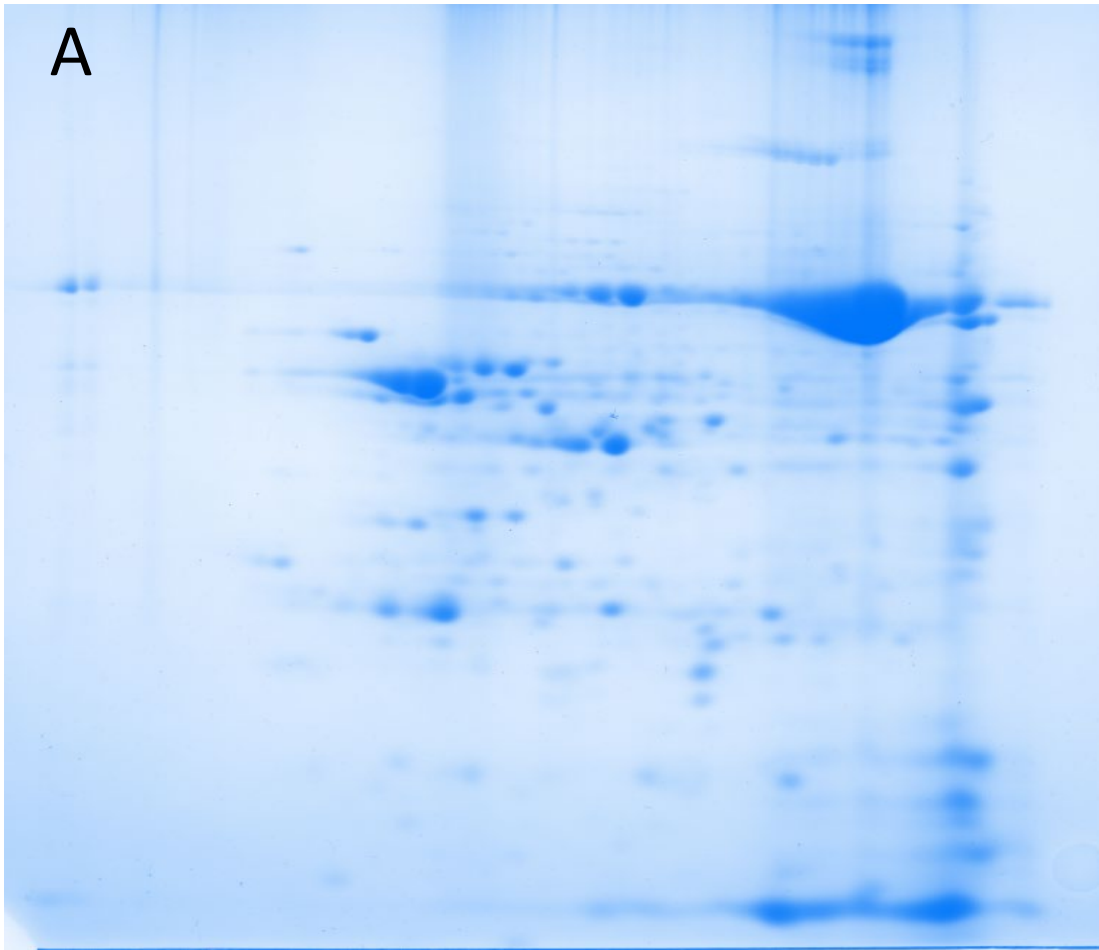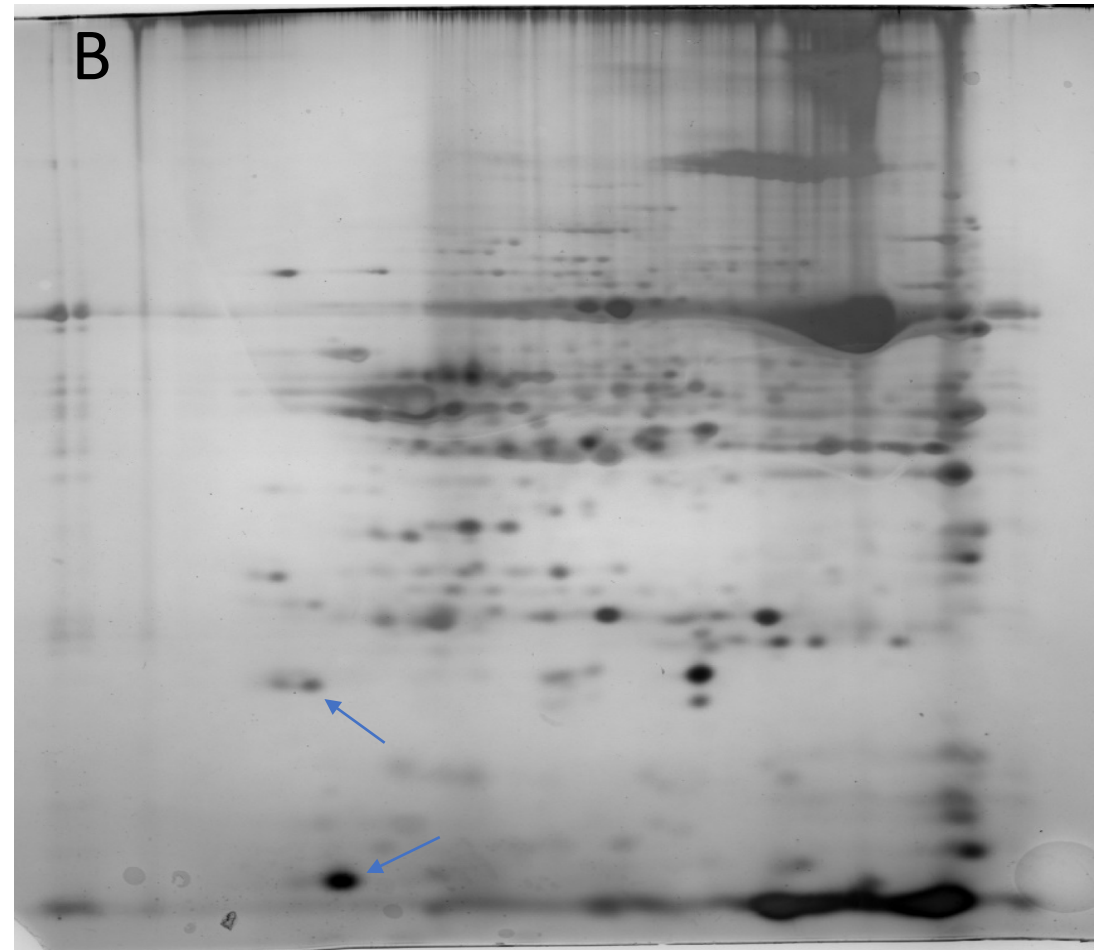

Supplement: Supplementary Figure 2 — Comparison of staining method in 2 D Gel analysis of control rice leaves samples (A) Colloidal coomassie staining and (B) Silver staining. The arrow indicates the protein spots detected only in silver staining, not coomassie staining. [file Image_2.pdf]
